# Metagenome-assembled genomes of four psychrotolerant *Clostridium*-affiliated bacteria from an Fe(III)-reducing enrichment culture of Antarctic penguin-impacted soil

**DOI:** 10.1128/mra.00854-26

**Published:** 2026-10-13

**Authors:** Xin Wang, Hongyan Wang, Jiaojiao Yang, Hong Chen, Xiaodong Liu, Xin Chen, Li Liao, Yiran Dong, Lian Yu, Zongming Xiu, Yi Yang

**Affiliations:** 1 Key Laboratory of Forest Ecology and Silviculture, Institute of Applied Ecology, Chinese Academy of Sciences, Shenyang, Liaoning, China; 2 University of Chinese Academy of Sciences 74519, Beijing, China; 3 Water Resources Research Center, University of Hawaii at Manoa, Honolulu, Hawaii, USA; 4 Department of Civil, Environmental and Construction Engineering, University of Hawaii at Manoa, Honolulu, Hawaii, USA; 5 Anhui Province Key Laboratory of Polar Environment and Global Change, School of Earth and Space Sciences, University of Science and Technology of China, Hefei, Anhui, China; 6 Key Laboratory for Polar Ecosystem and Climate Change of Ministry of Education, School of Oceanography, Shanghai Jiao Tong University 12474, Shanghai, China; 7 Shanghai Key Laboratory of Polar Life and Environment Sciences, Shanghai Jiao Tong University 12474, Shanghai, China; 8 School of Environmental Studies, China University of Geosciences (Wuhan), Wuhan, China.; 9 Department of Environmental Engineering, Beijing Institute of Petrochemical Technology 34735, Beijing, China; 10 Zhejiang Key Laboratory of Environment and Health of New Pollutants, School of Environment, Hangzhou Institute for Advanced Study, University of Chinese Academy of Sciences, Hangzhou, China; Montana State University, Bozeman, Montana, USA

**Keywords:** *Clostridium*, Fe(III)-reducing bacteria, Antarctic, Psychrotolerant

## Abstract

We report four *Clostridium*-affiliated metagenome-assembled genomes (MAGs) recovered from a psychrotolerant enrichment culture capable of Fe(III) reduction at 4°C, seeded from Antarctic penguin-impacted soil. The four MAGs ranged from 3.6 to 5.3 Mb in size, with G + C contents of approximately 31.0% and estimated genome completeness ranging from 82.76% to 98.54%.

## ANNOUNCEMENT

The four *Clostridium*-affiliated MAGs designated Antarctic1, Antarctic2, Antarctic3, and Antarctic4 were reconstructed from the metagenome of a mixed anaerobic Fe(III)-reducing enrichment culture. This culture was derived from ornithogenic (penguin-impacted) soil collected aseptically in 2010 near active emperor penguin nesting sites on Beaufort Island, Antarctica (166°58′23.6″E, 76°58′23.6″S) at a depth of 17.5 cm. The sampled soil profile consisted of a fine-grained, black layer enriched by penguin guano overlying brown clay. Samples were collected using disinfected tools, immediately frozen, and maintained at −80°C until enrichment experiments commenced in 2021. Approximately 5 g of homogenized interior soil material was used to establish the initial anaerobic microcosms (1). Cultivation was performed in triplicate 120-mL serum bottles containing 80 mL of bicarbonate-buffered reduced mineral salts medium (30 mM bicarbonate, pH 7.2) under a 40-mL N₂/CO₂ (80:20, vol/vol) headspace (2–4). The medium was amended with 10 mM sodium lactate, 0.5 g L⁻¹ peptone, and approximately 4 mM Fe(III) citrate. The bottles were incubated statically at 4°C in the dark. Following an initial incubation of approximately 3 months, sediment-free enrichment cultures were maintained by transferring 3% (vol/vol) of the culture into fresh medium under the same conditions (1). The mixed enrichment culture converted the initial approximately 230 mg L⁻¹ Fe(III) to Fe(II) within 3 months, with no residual Fe(III) detected within the analytical resolution of the ferrozine assay. The culture also produced a black precipitate subsequently identified as magnetite (1). Because these genomes were reconstructed from a mixed consortium rather than obtained as individual axenic isolates, the observed Fe(III) reduction cannot be definitively attributed to a single strain. Nevertheless, these MAGs provide critical genomic insights into cold-adapted fermentative iron reduction, a process that remains poorly understood in extreme polar environments.

Biomass was harvested from 1 mL of the mixed fifth-transfer Fe(III)-reducing enrichment culture by centrifugation at 13,000× *g* for 30 min at 4°C. Metagenomic DNA was extracted from the resulting cell pellet using the modified sodium dodecyl sulfate (SDS) method described previously (5). DNA quality was verified by gel electrophoresis, and DNA concentration was measured using a Qubit 2.0 fluorometer (Thermo Scientific). Metagenomic sequencing was performed on the Illumina NovaSeq 6000 platform (6). Raw reads were filtered using Readfq V8 and assembled using the JGI Metagenome Assembly Pipeline (7). Read-level taxonomic classification was performed using Kaiju v1.7.3 (8), and metagenomic contigs were binned using MaxBin2 v2.2.4 (9). MAG completeness and contamination were assessed using CheckM v1.1.3 (10). Taxonomic assignments were determined using GTDB-Tk v2.3.2 and TYGS (11, 12). Functional annotation was performed using DRAM, with genes called by Prodigal and compared against the Kyoto Encyclopedia of Genes and Genomes (KEGG), UniRef, Pfam, and dbCAN databases using DIAMOND and HMMER. The deposited genome assemblies were additionally annotated using the National Center for Biotechnology Information (NCBI) Prokaryotic Genome Annotation Pipeline (PGAP) v6.10 (13). Default parameters were used unless otherwise specified.

Sequencing generated 35,220,421 paired-end read pairs (70,440,842 individual reads; 10,566,126,300 total bases). The draft genomes of Antarctic1–4 consisted of four MAGs with estimated genome sizes ranging from 3.6 to 5.3 Mb and guanine + cytosine (G + C) contents of 31.0% (Table 1). The assemblies contained between 42 and 95 contigs and encoded 3,253 to 4,986 coding DNA sequences (CDSs). Genome completeness estimates ranged from 82.76 to 98.54%, with contamination levels below 4%. MAG quality was classified according to the Minimum Information about a Metagenome-Assembled Genome (MIMAG) standards (14). Based on these criteria, Antarctic1 and Antarctic3 were classified as high-quality draft MAGs, whereas Antarctic2 and Antarctic4 were classified as medium-quality draft MAGs. GTDB-Tk identified *Clostridium* sp. 016650035, *Clostridium lacusfryxellense* DSM 14205, *Clostridium bowmanii* DSM 14206, and *Clostridium tagluense* A121 as the closest references to Antarctic1, Antarctic2, Antarctic3, and Antarctic4, respectively, with average nucleotide identity (ANI) values ranging from 84.71 to 90.57%. These ANI values are reported as descriptive comparisons and are not interpreted as evidence of phylogenetic distinctness or taxonomic novelty. In addition, multiple cold shock proteins (Csp) and hydrogenase-associated genes were identified in all four MAGs, indicating potential genomic adaptations for low-temperature survival and hydrogen-based metabolism in anoxic polar environments. Collectively, these MAGs expand the genomic representation of *Clostridium*-affiliated bacteria recovered under low-temperature, anoxic conditions and provide genomic resources for future investigations of microbial metabolism in Antarctic terrestrial environments.

**TABLE 1:** Genomic characteristics of four MAGs reconstructed from an Fe(III)-reducing enrichment culture established with Antarctic penguin-impacted soil

Feature	Antarctic1	Antarctic2	Antarctic3	Antarctic4
Genome size (Mb)	4.4	3.6	4.5	5.3
G + C content (%)	31.0	31.0	31.0	31.0
No. of contigs	42	46	95	42
N50 (kbp)	253.5	136.0	73.8	194.4
Coding sequences (CDSs)	4,173	3,253	4,116	4,986
5S/16S/23S rRNA genes	12/5/2	7/0/1	7/3/5	6/1/1
No. of tRNA genes	92	72	62	75
NCBI assembly accession	GCF_050717635.1	GCA_050717545.1	GCA_050717535.1	GCA_050717515.1
BioSample accession	SAMN45115834	SAMN45115835	SAMN45115836	SAMN45115837
Completeness (%)	98.54	97.09	94.48	82.76
Contamination (%)	3.81	2.04	1.77	3.45
Closest GTDB reference	*C*. sp. 016650035	*C. lacusfryxellense* DSM 14205	*C. bowmanii* DSM 14206	*C. tagluense* A121
Reference genome accession	GCA_016650035.1	GCF_018861735.1	GCF_018861315.1	GCF_003865095.1
ANI to closest reference (%)	90.3	84.71	90.32	90.57
MIMAG quality category	High	Medium	High	Medium
Raw metagenomic sequencing output^*a*^	35,220,421 paired-end read pairs (70,440,842 reads; 10.57 Gbp)			

^*a*^ All four MAGs were reconstructed from the same mixed-enrichment metagenomic sequencing data set.

## Data Availability

The MAGs Antarctic1–4 have been deposited in GenBank under the accession numbers GCA_050717635.1, GCA_050717545.1, GCA_050717535.1, and GCA_050717515.1. The raw metagenomic sequencing reads have been deposited in the Sequence Read Archive under accession number SRR38308589. The corresponding BioSample accession numbers are SAMN45115834, SAMN45115835, SAMN45115836, and SAMN45115837 under BioProject accession number PRJNA1192582.
